# Incidental Finding of Thyroid Tuberculosis by Operation for Graves' Disease: A Rare Case Presentation

**DOI:** 10.1155/2024/3865608

**Published:** 2024-03-06

**Authors:** Zineb El Azime, Samira Handa, Ghita Bourkadi, Hayat Aynaou, Houda Salhi, Hanan El Ouahabi

**Affiliations:** ^1^Department of Endocrinology, Diabetology and Nutrition, Hassan II University Hospital, Fez, Morocco; ^2^Epidemiology and Health Sciences Research Laboratory, Fez, Morocco; ^3^Faculty of Medicine, Pharmacy and Dental Medicine, Sidi Mohamed Ben Abdellah University, Fez, Morocco

## Abstract

Thyroid tuberculosis is a rare form of extrapulmonary tuberculosis, even in endemic countries such as Morocco; its precise incidence is not well-defined. The clinical presentation of thyroid tuberculosis can vary, often being asymptomatic. Consequently, the diagnosis may be overlooked or delayed. Intriguingly, thyroid tuberculosis can manifest alongside thyrotoxicosis due to Graves' disease, an exceptional association. This article reports a distinctive case of thyroid tuberculosis coexisting with Graves' disease, one of the autoimmune thyroid diseases. A 33-year-old female presented symptoms consistent with hyperthyroidism and bilateral exophthalmos. She was subsequently diagnosed with Graves' disease. Despite initially managed medically, recurrences of the disease led to definitive treatment by total thyroidectomy. Histopathological examination revealed concurrent presence of thyroid tuberculosis.

## 1. Introduction

Thyroid tuberculosis is an exceedingly rare condition, even among populations with a high prevalence of tuberculosis [1, 2]. It was first reported by Lebert in 1862 as part of a study on disseminated tuberculosis [3]. Extra-pulmonary tuberculosis, when compared to pulmonary tuberculosis, can present with varied clinical manifestations, and can pose significant diagnostic challenges.

While thyroiditis, nodules, cysts, abscesses, and carcinoma of the thyroid gland have all been associated with thyroid tuberculosis, the concomitant presence of Graves' disease with thyroid tuberculosis is notably unusual. In this article, we present a remarkable case not only for the co-occurrence of these two conditions but also because it represents a rare instance of primary thyroid tuberculosis.

## 2. Case Presentation

A 33-year-old woman, with no significant medical history, presented with symptoms of hyperthyroidism: palpitations, asthenia, and a weight loss of 6 kg within a month. She also had bilateral exophthalmos that persisted for 6 months. Clinical evaluation revealed a tachycardia of 120 beats/min. A physical examination of her neck showed a homogeneous goiter with an elastic consistency and pronounced vascularity.

Laboratory investigations showed an ultrasensitive TSH level of less than 0.05 mU/L. The serum-free T4 level was elevated at 43.24 pmol/L (reference range: 10–28.2 pmol/L), the serum-free T3 level was 13.33 pmol/L (reference range: 4–8.3 pmol/L), and anti-TSH receptor antibody was elevated at 24 IU/L (reference range: <1.5 IU/L). Cervical ultrasound demonstrated a thyroid gland with a homogeneous texture and pronounced vascularity on Doppler imaging. Based on these findings, the diagnosis of Graves' disease was made. Notably, her electrocardiogram, complete blood count, and liver transaminase levels were all within normal range.

**Figure 1:**
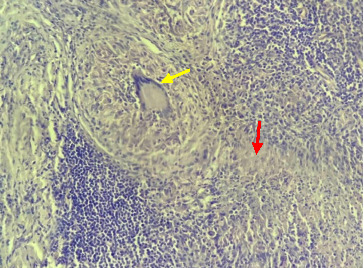
Histological sections (at ×40 magnification) showing thyroid parenchyma completely erased and replaced by epithelioid granuloma (yellow arrow) with caseous necrosis (red arrow).

**Figure 2:**
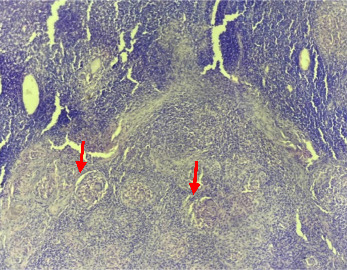
Histological sections (at ×40 magnification) showing multiple epitheloid granulomas.

The patient was initiated on carbimazole (30 mg/day) and propranolol (80 mg/day), which led to clinical improvement and normalization of thyroid function. However, after 18 months of marginally effective antithyroid treatment, the medication was discontinued, and the patient was closely monitored. She experienced two relapses, with a an average remission period of six months and after 18-month of antithyroid treatment, each associated with elevated anti-TSH receptor antibody levels (19 IU/L and 22 IU/L, respectively), increased serum-free T4 and T3 levels, and a homogeneously hypervascular goiter. Given these findings, a definitive treatment approach was discussed. The patient opted for a total thyroidectomy. Histopathological analysis of the excised thyroid tissue revealed complete erasure of the thyroid parenchyma, replaced by multiple epithelioid granulomas, as illustrated in Figures 1 and 2. In this case, the diagnosis of thyroid tuberculosis was confidently established based on the characteristic histopathological findings, additional diagnostic investigations such as special stains on the specimen or PCR for tuberculosis were not employed.

The patient had an uneventful postoperative period. Sputum tests for tuberculosis and chest X-ray were negative for any pulmonary lesions. She subsequently began a 6-month antituberculosis treatments regimen: an initial 2-month phase with isoniazid, rifampicin, and pyrazinamide, followed by 4 months of isoniazid and rifampicin (2HRZ/4HR). Lifelong replacement therapy with levothyroxine was also initiated, with an average daily dose of 100 *µ*g, equivalent to 0.97 *µ*g/kg/day. The patient's clinical course was marked by the resolution of thyrotoxicosis symptoms, resulting in a weight gain of 7 kg over 6 months and normalization of thyroid function tests.

## 3. Discussion

*Mycobacterium tuberculosis* (MTB) is an aerobic bacillus that causes most cases of tuberculosis. One-third of the world's population is estimated to be infected. [4]. With 40% of all cases occurring in India and China, Asia and Africa have the highest incidence [5]. Tuberculosis (TB) affects the lungs in 90% of cases [6]; however, extrapulmonary TB, which most frequently affects the lymph nodes [7], occurs in 15–20% of cases.

Even in areas where the prevalence of TB is very high, primary thyroid tuberculosis is an uncommon extrapulmonary manifestation of the disease. It is estimated that it occurs in 0.1–0.4% of all TB cases [8].

The underlying mechanisms of comparative high resistance of thyroid gland to TB infection are unknown. Primary TB at this site is uncommon, which may be attributed to several factors, including the thyroid capsule, high iodine levels within the gland, the colloid's bactericidal action, the thyroid hormones' antibacillar effects, and the thyroid gland's abundant lymphatic and vascular supply [9, 10].

We present an extremely rare case of thyroid tuberculosis in association with Grave's disease. The stimulation of thyroid TSH receptors by anti-TSH receptor antibodies increases thyroid hormone synthesis due to Graves' disease, these thyroid changes will cause vascular problems that make the thyroid vulnerable to bacillary attack [9]. During Graves' disease, the heightened production of thyroid hormone is typically responsive to the action of antithyroid drugs, which act by inhibiting thyroid peroxidase (TPO) and have an immunosuppressive effect [11, 12]. The lack of response to antithyroid drugs in the reported case raises the question of whether the Koch bacillus could modulate the effects of carbimazole or thyroid peroxidase, although this hypothesis is also not fully substantiated [9].

Thyroid tuberculosis remains difficult to diagnose without clinical or biological guidance (TB disease, history of tuberculosis, cutaneous fistula, mass lesion, nodule, and inflammatory syndrome). The presence of Graves's disease makes the clinical symptomatology more misleading. In our case the the lack of typical clinical signs and homogeneous ultrasound findings further complicates the diagnosis.

To make a preoperative thyroid tuberculosis diagnosis, imaging procedure such as thyroid scintigraphy is useful as it shows hypoactive areas in thyroid gland [13]. In our case, scintigraphy was not performed before surgery.

Confirmation through bacteriological testing of the presence of Koch bacillus in a thyroid or granuloma epithelioid gigantocellular with caseous necrosis during the histological examination of the piece of thyroidectomy is required for diagnostic confirmation.

Thyroid tuberculosis is treated medically, except for the complicated forms (abscess and fistulization). This therapy consists of combining potent antibacillary drugs [14]. In our situation, definitive treatment was recommended due to multiple relapses, the patient chose a complete thyroidectomy. The procedure serves both therapeutic and diagnostic purposes with a frequently observed positive outcome and minimal adverse effects. However, thyroidectomy is not without potential complications, and concurrent thyroid tuberculosis infections may be correlated with infections of other tissues, underscoring the importance of meticulous patient care and management.

Table 1 presents a summary of documented cases in the literature that specifically involve thyroid tuberculosis with hyperthyroidism, including our case (Table 1).

## 4. Conclusions

In conclusion, thyroid tuberculosis is a rare entity. The vulnerability of the thyroid to infection may increase with Graves' disease. Clinicians should consider the possibility of an association between thyroid tuberculosis and cases of hyperthyroidism relapse, particularly in regions where tuberculosis is endemic.

## Figures and Tables

**Table 1:** Summary of cases of thyroid tuberculosis in the literature.

Cases	Ersoy et al. [1]	Raman et al. [8]	Ouleghzal et al. [9]	Our case
Case number	1	1	1	1
Patient age	40 years	38 years	42 years	33 years
Gender	Female	Male	Female	Female
Clinical presentation	(i) Signs of hyperthyroidism (ii) Lymphadenopathy + thyroid nodules	(i) Signs of hyperthyroidism + nodular goiter + high fever	(i) Hyperthyroidism (ii) Bilateral exophthalmia (iii) Homogeneous goiter, elastic, and very vascular	(i) Signs of hyperthyroidism (ii) Homogeneous goiter, and a very vascular
Diagnostic tests	(i) TSHus: 0.0007 IU/ml (0.34–4.2) (ii) FT3: 7 pg/ml (2.5–3.9) (iii) FT4: 5.05 ng/dl (0.7–1.4) (iv) Thyroid scintigraphy: grave's disease + benign hypoactive nodule (v) Fine needle aspiration (FNA): caseification necrosis	(i) TSH: 0.14 mIU/L (ii) FT4: 25.1 ng/dL (iii) CRP: 373 mg/L (iv) ESR: 43 mm/h (v) Ultrasound and CT scans: multinodular thyroid enlargement (vi) Thyroid scintigraphy: no uptake in the thyroid	(i) TSH <0.005 mUI/ml (ii) FT4: 98 pmol/l (12−22 pmol/) (iii) FT3: 54 pmol/l (3,2–6 pmol/l) (iv) Anti TSH receptor Ab (TRAb): 7.6 mUI/ml (<2) (v) Cervical ultrasound: homogeneous hypo-echogenic thyroid that is highly vascularized on doppler	(i) TSH <0.05 mU/L (ii) FT4: 43.24 pmol/L (10–28.2) (iii) FT3: 13.4 pmol/L (4.0–8.3) (iv) TRAb: 24 IU/L (<1.5 IU/L) (v) Cervical ultrasound: Homogeneous thyroid, highly vascularized on doppler
Initial diagnostis	Grave's disease + TB	TB thyroiditis on fine needle aspiration (FNA)	Grave's disease resistant to treatment	Grave's disease with multiple relapses
Histological diagnostic	coexistence of TB and the graves' disease	—	coexistence of TB and the graves' disease	coexistence of TB and the graves' disease
Treatment approach	Medical treatment + thyroidectomy and TB treatment	TB treatment (6 months)	Medical treatment + thyroidectomy and TB treatment	Medical treatment + thyroidectomy and TB treatment
Outcome	Favorable outcome	Favorable outcome	Favorable outcome	Favorable outcome

*Note*. TSH—thyroid-stimulating hormone, FNA—fine needle aspiration, and TB—tuberculosis.

## Data Availability

The data supporting the findings of this study are available upon reasonable request from the corresponding author.
